# Multiplexed Optical Nanobiosensing Technologies for Disease Biomarker Detection

**DOI:** 10.3390/bios15100682

**Published:** 2025-10-09

**Authors:** Pureum Kim, Min Yu Choi, Yubeen Lee, Ki-Bum Lee, Jin-Ha Choi

**Affiliations:** 1 School of Chemical Engineering, Clean Energy Research Center, Jeonbuk National University, Jeonju 54896, Republic of Korea; 202450588@jbnu.ac.kr (P.K.); minyu5089@jbnu.ac.kr (M.Y.C.); ybdona@jbnu.ac.kr (Y.L.); 2 Department of Chemistry and Chemical Biology, Rutgers—The State University of New Jersey, Piscataway, NJ 08854, USA; kblee@rutgers.edu

**Keywords:** fluorescence detection, multiplex detection, nanomaterials, biomarkers, disease diagnosis

## Abstract

Most biomarkers exhibit abnormal expression in more than one disease, making conventional single-biomarker detection strategies prone to false-negative results. Detecting multiple biomarkers associated with a single disease can therefore substantially improve diagnostic accuracy. Accordingly, recent research has focused on precise multiplex detection, leading to the development of sensors employing various readout methods, including electrochemical, fluorescence, Raman, and colorimetric approaches. This review focuses on optical sensing applications, such as fluorescence, Raman spectroscopy, and colorimetry, which offer rapid and straightforward detection and are well suited for point-of-care testing (POCT). These optical sensors exploit nanoscale phenomena derived from the intrinsic properties of nanomaterials, including metal-enhanced fluorescence (MEF), Förster resonance energy transfer (FRET), and surface-enhanced Raman scattering (SERS), which can be tailored through modifications in material type and structure. We summarize the types and properties of commonly used nanomaterials, including plasmonic and carbon-based nanoparticles, and provide a comprehensive overview of recent advances in multiplex biomarker detection. Furthermore, we address the potential of these nanosensors for clinical translation and POCT applications, highlighting their relevance for next-generation disease diagnostic platforms.

## 1. Introduction

The early, accurate, and highly sensitive diagnosis of diseases is critical in ensuring effective treatment and predicting therapeutic outcomes [1]. Hence, detection biomarkers such as nucleic acids and proteins have been extensively investigated for the diagnosis of diseases, including cancer and viral infections. The polymerase chain reaction (PCR), which offers high analytical accuracy, represents the current gold standard for nucleic acid detection [2]. However, PCR analysis is time-consuming and requires trained personnel, limiting the applicability of this method in rapid and on-site diagnostics [3]. Notably, the recent global viral pandemic has highlighted the urgent need for point-of-care (POC) testing systems capable of providing fast and reliable results in the field [4]. Indeed, biomarker detection platforms integrated with portable devices have subsequently been increasingly reported in response [5,6,7]. These diagnostic approaches rely on specific recognition of target biomarkers and the subsequent conversion to a measurable signal. Complementary DNA sequences are typically employed for nucleic acid targets [8], while protein targets are recognized using antibodies [9] or aptamers [10]. Various methods are commonly adopted to generate signals that depend on a specific target, including fluorescence [11], colorimetry [12], Raman spectroscopy [13], and electrochemical approaches [14]. Furthermore, the integration of diverse nanomaterials has led to enhanced signal intensity and improved detection sensitivity in readout systems [15,16]. Additionally, portable readout devices, including lateral flow assay (LFA) strips and microfluidic chips, have further advanced the development of rapid and POC diagnostic systems [17,18].

Conventional biomarker detection platforms typically target a single biomarker; however, many biomarkers are associated with various diseases [19]. For example, cancer biomarker miR-21, involved in regulating apoptosis, exhibits abnormal expression levels in multiple cancers, including pancreatic, breast, lung, and prostate cancer [20,21,22]. In addition, the blood levels of carcinoembryonic antigen (CEA), a common biomarker for colorectal cancer (CRC), are elevated not only in CRC but also in patients with breast, lung, pancreatic, gastric, liver, and ovarian cancers [23]. Consequently, diagnosing a specific disease based on the detection of a single biomarker is challenging. Simultaneous detection of multiple biomarkers can improve diagnostic accuracy by reducing false positive and false-negative results, while also minimizing sample volume, analysis time, and overall cost [24]. Thus, such multiplex detection strategies are particularly well suited for POC applications [25]. Numerous studies have recently reported systems capable of simultaneously detecting two or more biomarkers. These biomarkers can be associated with a single disease or with multiple diseases share similar clinical symptoms [26,27,28,29].

Among various readout methods, optical approaches convert biological interactions into optical signals [30] and are widely used for rapid and highly sensitive early disease diagnosis and monitoring [31]. Likewise, the easy integration of these methods with portable platforms makes them highly suitable for POC applications [32]. Fluorescence, one of the optical methods, relies on materials that absorb light at specific wavelengths, become excited, and subsequently emit photons. Different materials exhibit distinct excitation and emission wavelengths, allowing for the measurement of emission energy following irradiation with a specific excitation wavelength [33]. Due to its low sensitivity, Raman detection is frequently combined with metallic substrates to achieve surface-enhanced Raman scattering (SERS), providing material-specific spectral fingerprints [34,35]. Conversely, colorimetric detection is based on target-induced color changes and promotes the advantage of easy visual observation [36]. These readout methods can be applied to multiplex detection of two or more biomarkers [9,17,37,38]. However, these optical-based readout methods often suffer from weak inherent signals, limiting sensitivity. Various studies have incorporated nanomaterials with unique optical properties to address these limitations [39]. Notable examples include noble metal-based nanoparticles such as gold (Au) and silver (Ag) [40], as well as two-dimensional (2D) materials, including graphene oxide (GO) and MXene (2D transition-metal carbides/nitrides), and silica-based nanoparticles [41].

Recent reviews have highlighted advances in optical biosensors for multiplex biomarker detection [42] and in high-performance nano biosensing technologies for future diagnostic needs [43], reflecting both rapid technological progress and remaining challenges in clinical translation. In this review, we focus on optical-based multiplex nanosensors for the simultaneous detection of multiple disease-associated biomarkers. We examine the nanomaterials used to enhance sensitivity and specificity and review examples of their application in practical detection systems. Finally, we present the limitations of current multiplex biomarker detection technologies and provide perspectives on future directions in this field.

## 2. Nanomaterials for Enhancing the Performance of Biosensors

Nanomaterials, owing to their inherent structural and physicochemical properties, have been reported to contribute to the amplification or stable transduction of weak signals generated during molecular recognition processes in biosensors [44,45]. Their intrinsically high surface-to-volume ratios allow for the dense immobilization of recognition elements, thereby improving target capture efficiency, enhancing detection sensitivity, and lowering the limit of detection [44,46]. On the basis of these advantages, nanomaterials are increasingly recognized as promising platforms for enabling not only highly sensitive and selective analyses but also multiplexed detection, rendering them attractive candidates for next-generation biosensor design [47]. Accordingly, this chapter focuses on representative nanomaterials, including noble metals, silica-based nanostructures, graphene oxide, and photonic crystals, and highlights their physicochemical properties as well as the specific mechanisms by which they enhance biosensor performance.

### 2.1. Noble Metals

Noble metals, such as gold, silver, and platinum (Pt), possess chemical stability, excellent electrical conductivity, and biocompatibility properties, and are widely employed in biosensing [48,49]. In the bulk state, noble metals exhibit remarkable optical and electronic characteristics, where collective oscillations of conduction electrons at the metal–dielectric interface couple with incident light to produce surface plasmon resonance (SPR) [49]. By contrast, when the dimensions of these metals are reduced to the nanoscale, electron oscillations are confined to the nanoparticle surface, resulting in localized surface plasmon resonance (LSPR), which enables strong light–matter interactions and enhanced electromagnetic fields in the vicinity of the nanostructure [50,51]. These unique plasmonic phenomena provide the basis for highly sensitive biosensing platforms by amplifying optical signals and enabling molecular-scale detection.

LSPR is highly sensitive to parameters such as particle size, shape, interparticle spacing, and changes in the local refractive index. Its excitation generates an intense localized electromagnetic field in the surrounding region, which can significantly enhance the excitation efficiency of nearby fluorophores [52,53,54]. This phenomenon, known as metal-enhanced fluorescence (MEF), increases the quantum yield and photostability of conventional fluorophores and markedly enhances the signal-to-noise ratio (SNR). The efficiency of MEF influenced not only by the distance between the metallic surface and the fluorophore [4,55], but also by additional factors such as the composition of the metallic nanostructure [56], the dielectric environment, and the degree of spectral overlap between the excitation/emission bands of the fluorophore and the absorption/scattering spectra of the nanostructure [56,57]. In particular, the distance between the fluorophore and the metallic surface plays a decisive role, with an optimal separation of ~7–8 nm enabling enhancement, while closer proximity can instead induce quenching through non-radiative energy transfer [37,56,57,58,59,60]. Consequently, precise control of this gap in applications is essential, and is often achieved by introducing dielectric spacers such as silica, Al_2_O_3_, or polyethylene glycol (PEG), as well as molecular linkers including DNA or polymers [61,62,63].

Gold, the most widely utilized noble metal, can be synthesized into various nanostructures such as nanorods, nanoshells, nanostars, and nanoclusters, each of which exhibits distinct LSPR characteristics. Specifically, gold nanoparticles typically exhibit a distinct LSPR band around 520 nm, which undergoes a red shift as particle size increases [64]. In addition, nanorods display two separate plasmon bands, consisting of a transverse band near 530 nm and a longitudinal band whose resonance gradually shifts toward longer wavelengths (approximately 600–1500 nm) with increasing aspect ratio [65]. This is particularly advantageous for simultaneous fluorescence detection [66,67,68]. Comparatively, nanostar structures generate multiple high-intensity electromagnetic “hotspots” at their sharp tips, leading to pronounced MEF effects [69,70,71,72,73]. In addition, another merit of gold is the gold-thiol chemistry, which allows for the stable immobilization of various recognition elements, such as ssDNA, aptamers, antibodies, and peptides, ensuring high selectivity and specificity toward diverse biological targets. These properties render Au-based nanomaterials essential for the design of platforms capable of sensitive biomarker detection in disease diagnostics [37,74,75,76,77].

Spherical silver nanoparticles typically exhibit an LSPR peak around 400 nm [78]. Compared to gold, silver nanomaterials provide superior electrical conductivity and a higher plasmon resonance frequency. Thus, silver nanomaterials can generate exceptionally strong LSPR fields and exhibit high MEF efficiency [79]. These properties make Ag-based platforms particularly advantageous for ultra-sensitive fluorescence detection and single-molecule analysis. Furthermore, the sensitivity and selectivity of Ag nanomaterials can be optimized through structural engineering into diverse morphologies such as nanocubes, nanoplatelets, and nanowires [80,81,82]. An enzyme-free fluorescence microarray employing aggregates of silver nanoparticles (AgNPs) was developed for the determination of hepatitis B virus DNA, where the plasmonic enhancement enabled a detection limit down to 50 fM with over 1500-fold signal amplification compared to unenhanced fluorescence [83]. However, due to the low surface stability of silver, additional strategies such as protective coatings or alloy formation are often required. Nevertheless, Ag-based nanostructured platforms have demonstrated outstanding performance in a wide range of applications, including virus and bacterial detection, environmental monitoring, and clinical diagnostics [84,85].

Platinum nanomaterials not only exhibit excellent chemical and electrochemical stability and remarkable catalytic activity but can also be employed in optical biosensing due to intrinsic plasmonic properties that arise from their tunable nanostructures [86,87]. Pt is generally known to exhibit an LSPR peak in the UV region, although this can be tuned through morphological engineering [88]. Meanwhile, Au–Pt and Ag–Pt hybrid architectures synergistically combine plasmonic and catalytic functionalities, resulting in superior biosensing performance [89,90]. Although the synthesis of Pt nanostructures can be relatively complex and costly, the high sensitivity and selectivity of these nanostructures make them highly effective analytical platforms for a wide range of targets, including cancer metabolites, cardiovascular biomarkers, and infectious disease markers [91,92].

### 2.2. Silica-Based Nanomaterials

Silicon-based nanomaterials, such as silicon nanowires (SiNWs) and silicon nanoparticles (SiNPs), have attracted considerable attention in various biosensing platforms owing to their excellent optical and electrical properties as well as outstanding biocompatibility. Nanostructured porous silicon materials (PSi), including SiNWs and SiNPs [93,94,95,96], possess ultrafine dimensions that provide a large surface area and a high surface-to-volume ratio, thereby enabling the immobilization of recognition elements such as DNA, aptamers, and antibodies [97,98]. SiNWs, owing to their one-dimensional (1D) nanostructure, exhibit optical confinement and waveguiding effects that enhance the excitation and emission efficiency of fluorophores [93]. Furthermore, SiNWs, although not inherently highly conductive compared to metallic nanostructures, can exhibit enhanced electrical conductivity when appropriately doped or surface engineered. Such tunable electrical properties, combined with their optical waveguiding capability, make SiNWs attractive for the design of electro-optical hybrid biosensors [94,95,99]. Comparatively, SiNPs enable fluorescence emission through a size-dependent bandgap and can be combined with organic dyes or quantum dots to produce multi-channel fluorescence signals [96,100,101]. PSi structures, featuring nano- to micro-scale pores, facilitate analyte preconcentration and reduce photon reabsorption within the porous matrix, thereby improving fluorescence contrast [102,103,104]. A ratiometric fluorescence probe based on SiNPs was designed for visual detection of riboflavin (VB_2_), achieving a detection limit of 135 nM and enabling smartphone-assisted quantitative analysis with stable performance under diverse conditions [105].

A notable advantage of silicon-based nanomaterials lies in their surface functionalization via silane chemistry. Functional groups such as amine (–NH_2_), carboxyl (–COOH), and thiol (–SH) can be introduced to precisely control the immobilization of biomolecules, including DNA, proteins, and peptides [97,102,106,107]. This functional versatility enables selective binding and fluorescence readout not only for single biomarkers but also for multiplex biomarker panels [98,104,108]. Collectively, silicon-based nanostructures offer synergistic benefits in fluorescence signal enhancement, target enrichment, and multi-channel detection, underscoring the utilization of these structures in advanced biosensing applications.

### 2.3. Carbon-Based Nanomaterials

GO is a 2D nanomaterial derived from graphene, in which an sp-bonded carbon network is heavily decorated with oxygen-containing functional groups such as epoxide, hydroxyl, and carboxyl. This structural modification imparts a combination of large specific surface area, mechanical stability, excellent electrical and thermal properties, and outstanding surface chemical tunability [109,110,111]. The oxygen-containing groups on the GO surface confer hydrophilicity and a net negative charge [112], enabling electrostatic interactions, hydrogen bonding, and π–π stacking with numerous biomolecules, including nucleic acids, proteins, peptides, and antibodies [113].

This allows for the immobilization of diverse biorecognition elements onto a single GO substrate, facilitating the creation of parallel analytical channels for multiple targets within a single chip or sheet [114]. Notably, GO enables the spatially resolved immobilization of distinct probes on the same sheet, generating unique, target-specific signals for each channel. Furthermore, the high surface area of GO favors target preconcentration [115,116], where surface-bound probes efficiently capture low-abundance analytes from complex samples, thereby improving detection sensitivity. Furthermore, GO is an effective fluorescence quencher due to its energy transfer and non-radiative dipole–dipole coupling [117,118]. These properties render GO suitable not only for single-biomarker detection but also for simultaneous diagnosis, a critical requirement in the analysis of complex disease states [116,119]. In Wu et al.’s study, capture antibodies were immobilized on GO surfaces to simultaneously detect ovarian cancer biomarkers, cancer antigen 125 (CA125), human epididymis protein 4 (HE4), α -fetoprotein (AFP) and CEA as Raman signals [119].

Carbon nanotubes (CNTs) are one-dimensional carbon nanomaterials with a cylindrical structure of hexagonal graphene sheets, characterized by high electron mobility, excellent mechanical strength, wide specific surface area, and electrochemical stability [120,121]. However, the limitations of diameter and chirality control in the synthesis process, lack of dispersibility due to hydrophobicity, and concerns about biotoxicity are pointed out as disadvantages [122]. To overcome these limitations, surface functionalization techniques such as catalyst design-based control synthesis, surfactant and polymer enhancement, and PEGylation are being actively studied [122,123]. CNTs have a large specific surface area and excellent biomolecule adsorption capacity, which can efficiently immobilize various recognition molecules such as DNA, proteins, and antibodies [120,124]. In the study by Hu et al., horseradish peroxidase (HRP) and alkaline phosphatase (ALP) were modified into active reactants via click chemistry and subsequently self-assembled into enzyme aggregates, denoted as HRPn and ALPn, respectively. These enzyme aggregates were immobilized onto CNTs, which served as catalytic platforms to trigger chemiluminescence upon substrate conversion, enabling the simultaneous detection of AFP and Golgi protein 73 (GP73). The CNTs provided a large surface area and allowed for precise surface functionalization, thereby facilitating stable enzyme immobilization [123].

CNTs can function as a uniform immobilization substrate for polymer or metal nanoparticles based on their large specific surface area and excellent conductivity [125,126]. In particular, these metal-carbon hybrid structures greatly amplify Raman signals by increasing the density of local electromagnetic field hot spots [127]. Single-walled CNTs (SWCNTs) can emit light in the near-infrared (NIR) region, making them suitable for fluorescence imaging and detection in conditions with high biological tissue [128,129]. In addition, CNTs also act as efficient quenchers, and are used to manufacture Förster resonance energy transfer (FRET)-based “on/off” fluorescence sensors [130,131].

### 2.4. Photonic Crystal

Photonic crystal (PhC) beads are three-dimensional (3D) nano-microstructured materials comprising periodic refractive index variations that selectively reflect and transmit specific wavelengths of light, exhibiting unique optical properties [132,133]. Unlike metallic nanoparticles, whose optical enhancement relies on surface plasmon effects, PhC beads operate on the principle of photonic bandgap formation, enabling the manifestation of structural colors and the retention of intrinsic wavelengths with no changes in an external light source [134]. PhC beads are typically fabricated via the self-assembly of monodisperse spherical particles, where the highly ordered internal arrangement determines wavelength selectivity. Indeed, the structural color can be finely tuned to correspond to specific wavelengths by precisely controlling bead diameter, refractive index contrast, and periodic lattice spacing [135]. These wavelength-specific optical properties are exploited in multiplex analysis for both color-based coding and fluorescence signal enhancement [136]. The surface of the PhC beads can be chemically modified through salinization, polymer coatings, or introducing functional groups such as carboxyl and amine moieties, enabling the immobilization of diverse biomolecules, including oligonucleotides, proteins, and antibodies [137].

This functional versatility allows for the creation of bead libraries containing multiple unique structural color–fluorescence combinations, with each bead type conjugated to a probe specific for a particular biomarker. In multiplex detection, these bead types can operate in parallel, generating distinct fluorescence outputs that can be cross validated with their structural color signatures, significantly enhancing analytical reliability [138]. Additionally, the porous surface and internal architecture of PhC beads provide analyte preconcentration effects, improving detection sensitivity. In particular, a photonic crystal biosensor incorporating an eye-shaped cavity was engineered for the precise identification of cancer cells. This design, based on a square lattice of silicon rods, achieved a remarkably high quality factor, strong sensitivity in monitoring refractive index changes, and nearly perfect transmission efficiency, collectively underscoring its effectiveness for biomedical diagnostics [139]. When integrated into POC testing platforms, PhC beads enable rapid, efficient, and high-throughput multiplexed analysis [140].

These diverse nanomaterials, with their unique optical and electrical properties and excellent surface functionalization capability, can be integrated with readout modalities, such as fluorescence, Raman spectroscopy, and colorimetry, to create platforms capable of precise and selective biomarker detection. Such technologies can be incorporated into rapid POC diagnostic systems, thereby playing a pivotal role in various clinical applications, including early disease prediction and real-time monitoring.

## 3. Fluorescence-Based Multiplex Detection

Fluorescence is a photophysical process in which a molecule absorbs photons of a specific wavelength from an external light source and transitions to an excited electronic state. Subsequently, the molecule returns to the ground state by emitting light at a longer wavelength within a short timescale [141]. This phenomenon, characterized by the wavelength difference between excitation and emission, known as the Stokes shift, plays a pivotal role in improving the SNR in fluorescence-based sensors [142]. Owing to these fundamental optical properties, fluorescence has emerged as a powerful tool for highly sensitive and selective detection, enabling real-time and noninvasive monitoring in various biosensing applications, particularly for disease biomarkers [143].

Fluorescent particles are widely used in multiplex detection due to their high selectivity and the ability to emit at distinct wavelengths, allowing signals from different targets to be distinguished [144,145,146,147]. Various fluorescent materials employed in fluorescence-based sensors generally exhibit narrow emission bands, broad excitation ranges, and high photostability. These physical properties help minimize interference between multiple signals and enhance the accuracy of the analysis [41,148,149,150].

Importantly, the intensity and selectivity of the fluorescence signals can be modulated by the surrounding environment and the physicochemical properties of incorporated nanomaterials [151]. Engineered nanomaterials have been developed to enhance fluorescence performance through their distinctive optical properties, including LSPR and energy transfer efficiencies. These nanomaterials can significantly influence the excitation and emission dynamics of fluorescent molecules depending on the design, either enhancing the fluorescence intensity or inducing quenching effects [60,152,153]. Fluorescence-based sensing techniques offer significant advantages for the multiplexed detection of biomarkers, and the subsequent integration with nanomaterials further enhances performance, making these techniques the most widely reported approach for multiplexed nanosensors. In this section, we introduce commonly used fluorescent materials and fluorescence-based multiplex biosensing methods, along with their key characteristics.

### 3.1. Fluorescent Particles for an Effective Biosensing System

Various fluorescent materials have been widely employed in numerous studies, including quantum dots (QDs) [154], upconversion nanoparticles (UCNPs) [155], and carbon- or graphene-based nanomaterials such as carbon quantum dots (CQDs) and graphene quantum dots (GQDs) [156]. Each fluorophore possesses a distinct emission spectrum, which is crucial for tailoring their use in biosensing. For example, fluorophores emitting in the NIR region exhibit reduced tissue scattering and absorption, thereby enabling deep tissue penetration. In addition, spectrally well-separated emission peaks allow for simultaneous use of multiple fluorophores, each conjugated to a specific probe, thereby facilitating selective biomarker recognition in multiplexed detection.

QDs are semiconductor nanocrystals with dimensions on the order of a few nanometers, characterized by quantum confinement effects that promote unique optoelectronic properties distinct from their bulk counterparts [157]. These nanomaterials typically exhibit high quantum yield, excellent photostability, broad excitation spectra, and narrow emission bandwidths. Such properties make them ideal fluorescent probes for multiplexed biosensing with minimal signal interference and high spectral resolution [158,159]. A tunable emission wavelength represents one of the most advantageous features of QDs and can be precisely controlled by adjusting the size and composition of the particles. Thus, emission can be promoted across a wide spectral range, from the visible to the NIR region [160]. Recently, QDs that emit in the NIR range (NIR-QDs) have been developed to reduce the impact of tissue autofluorescence and to improve photon penetration into deeper biological tissues. These NIR-QDs have been successfully applied for the ultrasensitive detection of low-abundance biomarkers, including microRNAs (miRNAs), tumor-associated proteins, and circulating tumor DNA (ctDNA) [161,162,163,164,165]. Moreover, QDs possess a highly versatile surface chemistry that allows for facile functionalization with various biorecognition elements, such as antibodies, aptamers, and DNA probes. This facile functionalization facilitates the construction of biosensors with high target specificity and precision in biological environments, thus enhancing the overall analytical performance of fluorescence-based detection systems [166].

CQDs and GQDs are fluorescent nanomaterials derived from carbon, which are widely recognized for their excellent biocompatibility and low cytotoxicity. Although pristine CQDs and GQDs are not intrinsically water-soluble, their surfaces can be readily functionalized with oxygen-containing or other hydrophilic groups, imparting high aqueous dispersibility and stability [167]. These properties make CQDs and GQDs highly attractive for next-generation biosensing applications. Moreover, the photoluminescence characteristics of CQDs and GQDs can be finely tuned by controlling their size, surface functional groups, and dopant composition, allowing them to function as precise fluorescent encoding materials. CQDs offer superior photostability compared to traditional organic dyes and can produce turn-on or ratiometric fluorescence responses upon binding with diverse analytes such as metal ions or proteins, enabling robust quantitative sensing [156,168,169,170,171]. These capabilities have been utilized in CQD-based biosensors for the sensitive detection of various physiological signals, including pH changes, reactive oxygen species (ROS), and cancer-related proteins [169]. By leveraging the intrinsic 2D structure of graphene, GQDs exhibit strong π–π interactions that enable specific binding with biomolecules such as DNA, RNA, and proteins.

In addition, the emission properties of GQDs, which minimize spectral overlap with biological autofluorescence, together with their high signal-to-noise ratio, provide a favorable optical environment for highly sensitive measurements such as cellular imaging, FRET-based biosensing, and electrochemiluminescence assays [172,173]. Recently, GQDs have been integrated with cancer cell-specific aptamers, resulting in advanced biosensing platforms. These platforms can detect miRNAs at trace levels and gene mutations with high precision [174,175].

UCNPs are a class of fluorescent nanomaterials capable of absorbing two or more low-energy NIR photons sequentially and converting these photons into a single higher-energy photon, typically in the visible or ultraviolet range [176]. UCNPs are commonly engineered by doping rare-earth ions, such as Yb^3+^, Er^3+^, or Tm^3+^, into crystalline hosts such as NaYF_4_, NaGdF_4_, or NaLuF_4_. In these systems, Yb^3+^ typically acts as the sensitizer, absorbing NIR excitation, while Er^3+^ or Tm^3+^ serves as the emitter, responsible for the visible or UV emission [177]. Upconversion luminescence corresponds to an anti-Stokes shift, which contrasts with conventional Stokes emissions. Additionally, UCNPs exhibit several advantageous properties, including anti-Stokes shifts, deep tissue penetration, reduced background interference in their emission range due to minimal spectral overlap with tissue autofluorescence, and excellent photochemical stability [178,179]. Researchers can modulate key photophysical properties, such as emission wavelength, intensity, and lifetime, by tuning the dopant composition and concentration, thereby enabling the flexible design of detection systems for specific biological targets [180,181,182,183]. Notably, UCNP-based biosensors employing luminescence resonance energy transfer (LRET) mechanisms have demonstrated excellent performance in the ultrasensitive detection of biomarkers, including viral complementary DNA (cDNA) and miRNAs [184].

Furthermore, UCNPs are increasingly being applied in multiplexed biosensing platforms due to their distinct emission bands and low background interference, which offer capability within complex biological environments. Recent advances in fluorescence biosensing have increasingly incorporated nanomaterial-based signal enhancement strategies, such as LSPR amplification and the integration of nucleic acid amplification circuits, including catalytic hairpin assembly (CHA) and entropy-driven catalysis [185,186,187]. When coupled with nanomaterial platforms, these reaction circuits operate as autonomous molecular networks that continuously recycle target or reporter strands, thereby amplifying fluorescence signals without the need for enzymes and significantly improving sensitivity in multiplexed detection. These developments have led to the creation of highly sensitive, precise, and portable multiplex detection systems that meet the demands of modern clinical diagnostics.

### 3.2. Noble Metal-Enhanced Multiplex Fluorescence Nanosensor

Gold-based nanostructures possess a distinct advantage in enhancing fluorescence signals through LSPR, which, when combined with various fluorescent probes, makes these nanostructures widely applicable in multiplex detection platforms [51,188]. Structural variants such as nanospheres, nanorods, nanostars, and gold-coated magnetic particles offer diverse functionalities depending on their morphology [189,190]. This section highlights fluorescence-based biosensing approaches for multiplex detection of disease-related biomarkers, emphasizing fluorescence signal enhancement strategies employing diverse gold-based nanostructures and their distinctive characteristics.

**Figure 1:**
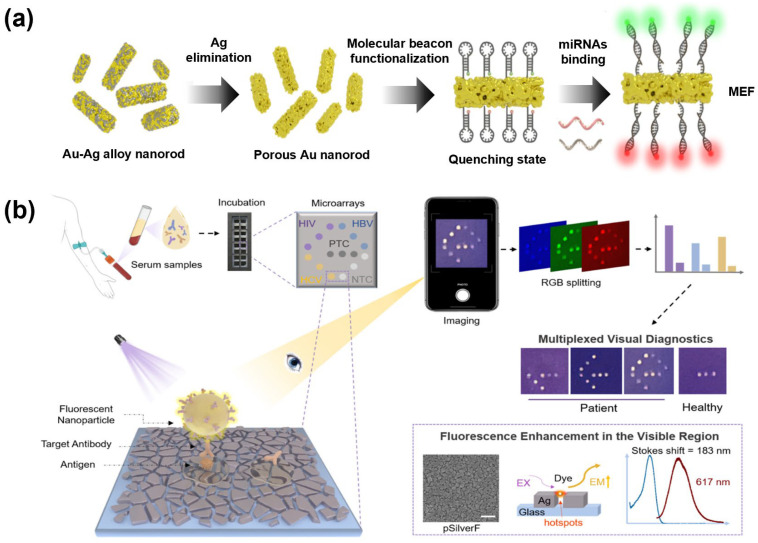
(**a**) Schematic illustration of a multiplex metal-enhanced fluorescence (MEF) biosensing platform using porous gold nanorods (pAuNRs). Molecular beacons labeled with distinct fluorophores were conjugated to pAuNRs, which induced MEF in the presence of their respective targets [37]. (**b**) Schematic of a portable naked-eye applicable multiplex diagnostic system based on a plasmonic-enhanced fluorescent nanoparticle biosensor for simultaneous detection of infectious diseases (HIV, HBV, and HCV) using PDDA-functionalized nanoparticles. Reprinted with permission from [191]. Copyright 2025, American Chemical Society.

Unlike conventional gold nanomaterials (smooth-surfaced), nanoparticles with rough surfaces exhibit an enhanced ultraviolet–visible (UV–Vis) spectrum over a broader range. Choi et al. developed a multiplexed detection system for miRNAs based on porous gold nanorods (pAuNRs) for the early diagnosis of cancer (Figure 1a). The pAuNRs were synthesized through the deposition of Au–Ag alloy and Ag etching, enabling broad-spectrum enhancement across the entire visible range. The sensing mechanism relied on simple hybridization between molecular beacons and their complementary target miRNAs, without the need for enzymatic amplification or complex sample preprocessing. Consequently, the platform successfully enabled the simultaneous signal enhancing and quantification of two cancer-related miRNAs, miR-21 and miR-141, with a 0.1 pM limit of detection (LOD) within one hour. The authors further noted that this pAuNR-based design holds strong potential not only for multiplexed early cancer diagnostics, but also for future adaptation to detect other nucleic acid biomarkers, including those derived from viral pathogens [37].

Tan et al. developed a plasmonic biosensor based on a novel plasmonic silver film (pSilverF) combined with a reporter to enable efficient MEF (Figure 1b). The pSilverF was fabricated by attaching Au seeds onto a glass slide, followed by the growth of a silver film that formed densely packed nanoscale Ag islands with nanoscale gaps; PDDA refers to PT-10, one of the fluorophores previously developed by the authors. In this study, PDDA was encapsulated into polystyrene nanoparticles (PDDA-NPs) to concentrate and enhance fluorescence visibility. The hotspots generated within the pSilverF structure significantly amplified the fluorescence of PDDA-NP, yielding approximately a 77-fold increase in fluorescence intensity compared with conventional glass substrates. Using this platform, simultaneous detection of HIV, HBV, and HCV antigens was demonstrated, with LOD values of 0.168, 0.023, and 0.0032 NCU/mL, respectively. For clinical applicability, serum samples from 42 patients and 26 healthy individuals were tested, achieving 100% specificity and over 96% sensitivity for all three antigens. The clinical validation confirmed the capability of highly specific multiplex detection, demonstrating the potential value of this approach for public health monitoring [191].

Liu et al. developed a fluorescence-based multiplex biosensor utilizing gold nanoparticles (AuNPs) as a functional platform. Hairpin-structured DNA probes, each labeled with a fluorophore–quencher pair, were immobilized on the AuNP surface to specifically recognize miR-21 and miR-122 within exosomes. Upon hybridization with their target miRNAs, a toehold-mediated strand displacement reaction occurred, separating the quencher from the fluorophore and thereby activating fluorescence. This reaction was further amplified through an entropy-driven catalysis mechanism, in which the displaced strand enabled each target miRNA to initiate multiple cascaded reactions without enzymatic involvement. In parallel, a DNA logic circuit constructed from interlinked hairpin probes provided programmable recognition, ensuring that only correct target inputs generated detectable signals, thereby improving specificity. Operating under isothermal conditions and allowing for direct in situ detection of exosomal miRNAs without extraction, this platform is highly suitable for point-of-care diagnostics. In clinical evaluation with serum samples from hepatocellular carcinoma patients, the biosensor simultaneously detected miR-21 and miR-122 with 93.3% sensitivity, 93.3% accuracy, an AUC of 0.92, and a limit of detection ranging from 2.4 × 10^5^ to 6.8 × 10^6^ particles/μL [192].

Chin et al. developed a dual-enhanced fluorescence biosensor that integrates chemiluminescence-based signal amplification with plasmonic enhancement for the rapid and accurate early diagnosis of sepsis. The target biomarkers consisted of six cytokines (MCP-1, IL-6, IL-10, IL-3, IL-1β, and TNF-α) that reflect disease progression and immune response changes in septic patients. The sensing substrate comprised a large-area gold nanodimple surface decorated with AuNPs, designed to generate strong localized electromagnetic fields to maximize LSPR effects on proximal fluorophores. Cytokine-specific antibodies were immobilized at discrete sensing spots to construct a multiplex array, while tyramide signal amplification (TSA) was employed during analysis to enhance the fluorescence output further. Detection was conducted using a laser line-scanning fluorescence imaging system, enabling simultaneous quantification of all six cytokines in independent channels on a single chip. The platform demonstrated detection limits below 1 pg/mL for all six cytokines, and clinical evaluation with plasma samples (*n* = 20) successfully distinguished sepsis patients from healthy controls with 100% accuracy [193].

Thus, noble metals have been applied in various forms to fluorescence-based sensing platforms, enabling the sensitive multiplexed detection of biomarkers. Further improvements in sensing efficiency can be achieved through the controlled tuning of nanoparticle morphology and the enhancement in their stability.

### 3.3. Quantum Dot-Based Multiplex Fluorescence Nanosensor

Carbon-based nanomaterials have attracted considerable attention in fluorescence-based multiplexed biosensing due to their excellent photostability and biocompatibility [194,195]. The superior fluorescence quenching properties mean that GO is effectively employed in Förster resonance energy transfer (FRET)-based signal modulation and in minimizing nonspecific background signals. In contrast, carbon dots (CDs) and CQDs serve as multicolor fluorescent probes owing to their tunable emission wavelengths and high quantum yields, enhancing analytical accuracy in multiplex detection platforms [196,197,198,199,200,201].

Various approaches have been reported for using QDs as fluorescent probes, enabling sensitive detection through efficient quenching by GO or enhancing QD stability by incorporating other nanomaterials during synthesis.

**Figure 2:**
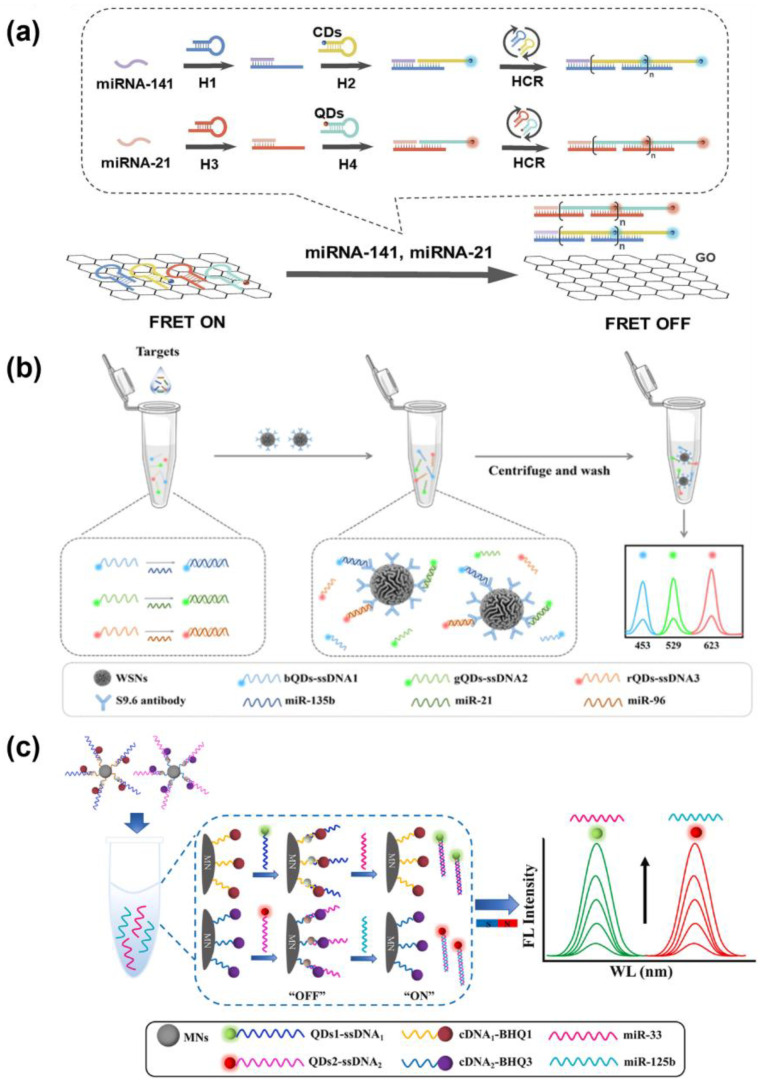
Fluorescence-based multiplex detection using QDs. (**a**) A schematic illustration of a FRET-based biosensor for simultaneous detection of miRNA-141 and miRNA-21 under a single excitation wavelength using CDs, QDs, and GO as donor–acceptor pairs. In the presence of miRNA-141 and miRNA-21, a hybridization chain reaction (HCR) triggered, leading to the release of DNA hairpins from the GO surface and recovery of fluorescence. Reproduced with permission from [202]. Copyright 2024, Elsevier. (**b**) A schematic representation of an amplification-free multiplexed biosensing platform for the detection of bladder cancer microRNAs targets (miR-135b, miR-21, and miR-96) are captured by antibody-modified WSNs, followed by hybridization with QD–ssDNA probes of different emission wavelengths (bQDs, gQDs, and rQDs). Reproduced with permission from [205]. Copyright 2024, American Chemical Society. (**c**) A schematic diagram of a DNA-functionalized double quantum dots-based fluorescence biosensor for one-step simultaneous detection. Each magnetic nanobead (MN) was linked to two black hole quenchers (BHQ1 or BHQ3) via complementary DNA (cDNA). In the presence of targets miR-33 and miR-125b, the formed duplexes were released into the supernatant after self-separation, restoring the fluorescence signal. Reproduced with permission from [206]. Copyright 2021, Elsevier.

Liu et al. developed a FRET-based biosensing platform to simultaneously detect two miRNAs (miR-21 and miR-141) under single-wavelength excitation, using CDs or QDs as fluorescent reporters and exploiting GO-mediated quenching (Figure 2a). DNA probes immobilized on GO exhibit fluorescence quenching, which is reversed upon hybridization with the target miRNA. The FRET-based biosensing platform enables multiplex detection with LODs of 60 pM and 50 pM for miR-21 and miR-141, respectively, highlighting the potential of fluorescence-based detection strategies for sensitive bioanalysis [202]. Similarly, a GO-based FRET platform using CQD-labeled probes, which remain quenched on GO until target miRNAs, miR-21 and miR-141, trigger a toehold-mediated strand displacement, releasing the probes and restoring fluorescence. The system employs enzyme-assisted repetitive reactions, including DNA polymerase-mediated strand extension and nicking enzyme cleavage, to amplify signals, with both targets achieving LODs of 4.7 fM. By using CQDs with distinct emission wavelengths, the platform enables fluorescence-based multiplex detection, demonstrating high sensitivity and selectivity in biological samples without additional isothermal amplification [203]. Li et al. developed a dynamic fluorescence-based biosensor for real-time, simultaneous detection of intracellular miR-21 and miR-10b, which are overexpressed in approximately 80% of tumors. The platform comprises ultrasound-driven Au nanowire/graphene oxide (AuNW@GO) nanomotors functionalized with complementary multicolor QD-labeled ssDNA probes to the target miRNAs. In this system, QDs act as fluorescent reporters, while GO serves as a FRET quencher, with fluorescence initially suppressed and subsequently restored upon target hybridization. Distinct QD emission wavelengths allow for interference-free multiplex detection under single-wavelength excitation, while ultrasound propulsion enhances intracellular probe delivery, thereby accelerating detection and improving accuracy. The biosensor achieved rapid OFF–ON fluorescence switching within 15 min, with quantitative detection demonstrated for both targets in the range of 0.5 to 10 pM. Furthermore, the method successfully discriminated between tumor (A549) and normal (L02) cells, showing excellent agreement with qRT-PCR validation [204].

Cheng et al. developed a QD-based fluorescent nanobiosensor aimed at the early diagnosis of HCC, to enable the simultaneous quantitative detection of three major serum biomarkers, α-fetoprotein (AFP), dickkopf-related protein 1 (DKK1), and glypican-3 (GPC3), all within a single clinical sample. The sensor was constructed by conjugating antibodies specific to each biomarker with CdSe/ZnS core–shell QDs that emit at 525 nm, 585 nm, and 625 nm, respectively. This multiplexed platform allowed for distinct signal separation without spectral interference and enabled accurate multi-analyte detection under identical assay conditions. By utilizing the intrinsic advantages of QDs, such as strong photostability, large Stokes shifts, and narrow emission bandwidths, the system provided resolved fluorescence signals for each target. Quantitative analysis was performed by measuring changes in fluorescence intensity in three distinct emission channels (525, 585, and 625 nm), corresponding to QDs covalently conjugated to antibodies against AFP, DKK1, and GPC3. Clinical serum samples were used for validation, and the QDs were retained within the antigen–antibody immunocomplex through stable covalent conjugation. The biosensor achieved detection sensitivities of 0.625 ng/mL for AFP, 1.25 ng/mL for DKK1, and 2.5 ng/mL for GPC3, showing enhanced diagnostic performance compared to single biomarker systems [207]. Wang et al. developed a fluorescence-based multiplex biosensor for sensitive detection of bladder cancer-related miR-21, miR-155, and miR-210 without enzymatic amplification (Figure 2b). The platform uses wrinkled silica nanoparticles (WSNs) as cores, densely loaded with QD-labeled DNA probes, each with a distinct emission wavelength. Target miRNA hybridization with the ssDNA probes induces conformational changes, increasing the distance between QDs and the silica/DNA interface that initially caused fluorescence quenching, thereby restoring the signal. Multicolor QDs enabled interference-free multiplex detection under single-wavelength excitation, with detection limits of 5 fM for miR-135b, 19 fM for miR-21, and 8 fM for miR-96, thereby demonstrating high sensitivity and specificity in serum samples [205]. Hu et al. developed a one-step fluorescence biosensor for the simultaneous detection of multiple miRNAs, specifically miR-33 and miR-125b, by integrating ssDNA-labeled dual QDs as donors with quencher-functionalized multifunctional nanomaterials (Figure 2c). The sensing mechanism is based on FRET, where target miRNA hybridization with complementary ssDNA probes restores QD fluorescence. This strategy enabled rapid and quantitative detection with low background, high sensitivity, and excellent specificity with no enzymatic amplification, achieving detection limits of 0.09 nM for miR-33 and 0.02 nM for miR-125b. Importantly, the platform allowed for simultaneous monitoring of these targets in human serum samples and cell extracts, demonstrating strong clinical applicability. Moreover, the dual-target approach reduced false-positive rates compared to single-analyte detection, offering a practical and robust alternative for multiplexed miRNA diagnostics [206].

### 3.4. Photonic Crystal-Based Multiplex Fluorescence Nanosensor

Ji et al. developed a fluorescence-based multiplex immunoassay for the early diagnosis of ovarian cancer using porous silica-based PhC beads. The system employed dendritic silica nanoparticles (dSiO_2_) with high surface area for stable immobilization of three antibodies targeting CA125, CEA, and AFP. Fluorescent signal generation was enabled by using CdTe QDs as detection probes. Each PhC bead exhibited a distinct optical diffraction pattern, originating from the periodic arrangement of dendritic silica nanoparticles that generate structural color via Bragg diffraction. These patterns were measured by optical reflection spectroscopy and served as intrinsic barcodes for bead identification. By assigning each diffraction pattern to a specific antibody functionalization, the system enabled precise discrimination and simultaneous detection of the three biomarkers within a single reaction system. Through a CdTe QD-based sandwich-type immunoreaction, the assay achieved LODs of 0.52 ng/mL for AFP, 0.64 ng/mL for CEA, and 0.79 U/mL for CA125 [208]. Notably, the system demonstrated higher sensitivity and reliability compared to traditional silica colloidal crystal bead (SCCB)-based assays. This study highlights the potential of combining fluorescent QDs with porous silica-based photonic platforms for precise multiplexed biomarker diagnostics.

Zhang et al. developed a multiplex screening platform for HPV nucleic acids based on a bioinspired PhC barcode. The PhC barcode is derived from the self-assembly of monodisperse colloidal nanoparticles, offering advantages such as stability and negligible fluorescence background. To enhance the efficiency of immobilization on PhC particles, the particles were coated with polydopamine (PDA), which contains abundant catechol and quinone groups, enabling efficient DNA probe attachment. Moreover, the PhC particles exhibit distinct reflection peaks dependent on the diameter of the silica nanoparticles, allowing for the fabrication of structurally colored colloidal crystal barcodes with unique wavelengths of 418 nm (blue), 520 nm (green), and 652 nm (red). Interestingly, HPV16, HPV18, and HPV33 were simultaneously detected using this system, demonstrating a LOD of 0.025 pM, indicating a highly sensitive multiplexed screening platform [209].

Furthermore, the same authors recently reported a clustered regularly interspaced short palindromic repeats (CRISPR)–based multiplex detection system utilizing PhC barcodes. The CRISPR-Cas13a system recognizes a specific RNA target and triggers trans-cleavage of adjacent single-stranded RNA. By leveraging this, once CRISPR recognizes the target, the loop region of a hairpin probe attached to the PhC particle is cleaved, exposing the initiator sequence. This proceeds the HCR, leading to the accumulation of numerous fluorophore-labeled DNA hairpins (H1 and H2) on the PhC barcode, thereby enabling signal detection. This study encapsulated the PhC barcodes within porous hydrogels to confine targets and probes within a limited space, thereby enhancing collision efficiency and improving detection sensitivity. Additionally, the high surface area–to–volume ratio of the hydrogel significantly enhanced the loading capacity of fluorescent probes, thereby improving overall detection efficiency. Simultaneous detection was performed for the *SARS-CoV-2 nucleocapsid (N)* gene, Influenza A virus (IAV), and Influenza B virus (IBV). The fluorescence signal demonstrated high sensitivity, exhibiting a linear response over a target concentration range of 1 fM to 1000 pM, with a remarkably low LOD of 200 aM [210].

### 3.5. UCNP-Based Multiplex Fluorescence Nanosensor

**Figure 3:**
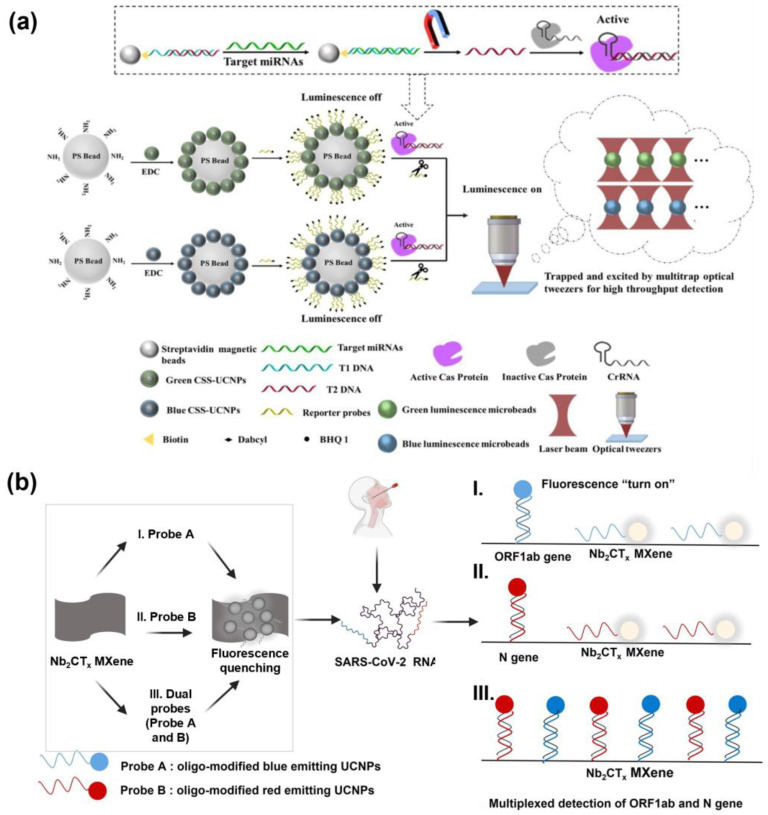
Multiplex biomarker detection systems using UCNPs. (**a**) Schematic illustration of an LRET-based biosensing platform for the simultaneous detection of dual miRNAs. Biotinylated T1 hybridizes with T2 on streptavidin-coated magnetic beads, where target miRNA displaces T2 via toe-mediated strand exchange. UCNPs are immobilized on PS beads using N-ethyl-N’-(3-(dimethylamino) propyl)-carbodiimide hydrochloride (EDC), and the activated CRISPR complex cleaves quencher-linked reporters to restore luminescence as the output signal. Reprinted with permission from [211]. Copyright 2024, American Chemical Society. (**b**) Schematic illustration of a UCNP/MXene-based fluorescence biosensing platform for multiplexed detection of SARS-CoV-2. UCNPs labeled with complementary oligonucleotides were noncovalently anchored onto MXene nanosheets, where their fluorescence was quenched via FRET. UCNPs hybridize with target RNAs to form duplex structures, leading to their desorption from MXene nanosheets and recovery of fluorescence signals. Reproduced with permission from [212]. Copyright 2022, Elsevier.

Yu et al. developed two types of luminescence-quenched UCNP–microbead assemblies that emit spectrally separated green (541 nm) and blue (475 nm) signals, enabling the simultaneous detection of dual miRNAs (Figure 3a). The selected UCNPs were engineered with activator ions confined to the inner shell, which minimized surface quenching and ensured stable emission with minimal spectral overlap, high photostability, and efficient LRET performance. Each UCNP–microbead assembly was functionalized with quencher molecules, which suppressed luminescence in the absence of targets. Upon the introduction of specific miRNAs, CRISPR–Cas12a was activated and cleaved the quencher-labeled reporters, thereby restoring the UCNP emission, with ~39% signal recovery even at concentrations as low as 2.5 fM. This Cas12a-mediated collateral cleavage served as an external signal amplification mechanism, since a single activated enzyme could remove multiple quenchers, yielding exceptionally low LODs of 17 aM and 22 aM for miR-155 and miR-let-7a, respectively. Furthermore, analysis of total RNA from cells demonstrated high correlation with qPCR results, and the method achieved single-cell-level sensitivity, underscoring the potential of the system for highly sensitive and specific biomarker detection [211].

Song et al. developed a fluorescence-based multiplex detection system that integrates UCNPs with MXene biosensor technology for the rapid and accurate diagnosis of COVID-19 (Figure 3b). This platform exploits the intrinsic anti-Stokes emission of UCNPs together with the large surface area and the high electrical conductivity of Ti_3_C_2_T_X_MXene, which facilitates efficient charge transfer and enhances its function as an effective fluorescence quencher in the FRET process, thereby improving the signal-to-noise ratio and enabling sensitive fluorescence responses to target concentration changes. Specifically, the system was designed to detect two target regions in the SARS-CoV-2 genome simultaneously, the *open reading frame 1ab* (*ORF1ab*) gene and the *N* gene, within a single reaction by employing distinct UCNP probes labeled with different fluorophores. In this design, UCNPs doped with Nd^3+^, Yb^3+^, Er^3+^, or Tm^3+^ absorb excitation at 980 nm and emit fluorescence at various wavelengths. Meanwhile, when conjugated with DNA probes, the target-specific hybridization could restore the fluorescence signals. Signal generation is based on the FRET interaction between MXene-induced fluorescence quenching and UCNP emission recovery, thereby minimizing background interference and achieving high precision. The detection limits were 15 fM for *ORF1ab* and 194 fM for the *N* genes, with excellent concordance demonstrated in clinical patient samples. This study highlights the potential of combining the unique optical properties of UCNPs with the stability of MXene surfaces to achieve fluorescence-based, highly sensitive, selective, and multiplexed dual-target detection [212].

Fluorescence-based multiplex biosensors have been extensively explored due to the diversity of fluorescent probes, the simplicity of assay design, and the advantages of signal amplification using nanomaterials. Furthermore, by integrating target-specific recognition elements such as nucleic acid probes, these platforms can be extended to cellular and in vivo sensing and imaging, enabling more precise real-time monitoring and therapeutic applications. UCNPs excited by NIR light offer a greater tissue penetration depth compared to conventional fluorophores, highlighting their significant potential for in vivo applications. A detailed list of representative fluorescence-based multiplex biomarker detection platforms is presented (Table 1).

## 4. SERS-Based Multiplex Detection

Raman spectroscopy provides molecularly specific vibrational information, but the inherently small scattering cross section (10^−30^ to 10^−25^ cm^2^) limits the applicability of Raman spectroscopy in trace analysis and single-molecule detection [213]. SERS exploits LSPR on metallic nanostructures to amplify Raman signals by factors ranging from thousands to millions, enabling the sensitive detection of low abundance molecules [214,215]. SERS-based multiplexed biosensors, which leverage the unique Raman scattering signatures of analytes, have emerged as highly selective diagnostic platforms capable of simultaneous biomarker detection [216,217].

An essential factor in SERS-based biosensor is the creation of hot spots, in which the electromagnetic field is significantly enhanced [218,219]. In general, many researchers have designed rough surfaces of nanomaterials or uniform nano gaps to facilitate the efficient formation of hot spots [220]. This approach can be applied to both substrate-based platforms and various forms of SERS tags, typically consisting of metallic nanoparticles and Raman reporters [38]. For target-specific detection, these nanostructures are further functionalized with biomolecules, enabling the generation of desired Raman signals only in the presence of target biomarkers. In this section, we introduce SERS-based biosensors that enable sensitive detection of multiple targets by generating hot spots through various nanostructured materials.

### 4.1. SERS-Based Detection Platform Using SERS Substrate

**Figure 4:**
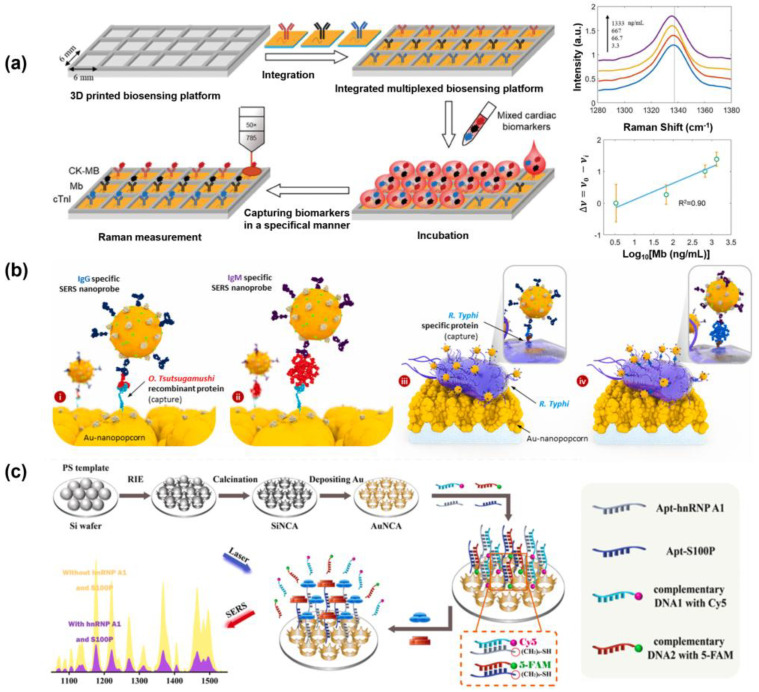
(**a**) A schematic diagram of a biosensor platform for multiple SERS detection of cardiac biomarkers and detection results of SERS frequency shift of target myoglobin are presented. The functionalized plasmonic metasurface was integrated into a 3D printed biosensing platform, and each row had the same type of monoclonal antibody functionalization. Different curve colors represent the SERS spectra of cardiac biomarkers at various concentrations. Reproduced with permission from [38]. Copyright 2024, Wiley. (**b**) Fabrication of gold nanopopcorn substrates and their integration into patterned microarray chips for SERS signal measurement of target antibodies. Color-coded nanoparticles (blue, purple, and red) represent distinct antibodies or pathogen-specific recognition events. And a schematic representation of a SERS-based serodiagnostic platform using plasmonic nanopopcorn microarrays for acute febrile diseases. Reproduced with permission from [221]. Copyright 2021, Elsevier. (**c**) A schematic illustration of the fabrication of SERS-based lab-on-a-chip (LoC) system (LoC-SERS) platform and sensing of multiplex detection of hnRNP A1 and S100P. Reproduced with permission from [8]. Copyright 2024, Elsevier.

Zheng et al. developed a highly reproducible SERS substrate for the multiplex detection of three major biomarkers associated with acute myocardial infarction (AMI) (Figure 4a). A gold–silica multilayered pyramidal plasmonic metasurface was fabricated to simultaneously enhance both the electric (E-field) and magnetic fields (H-field), thereby generating a dense distribution of hotspots over a large area. The metasurface was functionalized with specific Raman reporter molecules and monoclonal antibodies corresponding to each biomarker. Detection was achieved by monitoring the SERS frequency shifts induced by the presence of target biomarkers. Antibody–antigen interactions induce nanomechanical perturbations that alter the structure of Raman reporter molecules conjugated to the antibodies, resulting in characteristic frequency shifts in the SERS spectrum. Consequently, target-specific signal changes can be obtained using only individual monoclonal antibodies specific to each analyte, without the need for secondary labeling strategies. Specifically, 4-mercaptobenzoic acid (MBA), 5,5′-dithiobis-(2-nitrobenzoic acid) (DTNB), and 6-thioguanine (TG) were employed as Raman reporters to detect creatine kinase-MB (CK-MB), myoglobin (Mb), and cardiac troponin I (cTnI), respectively. The LODs were 0.04 ng/mL, 3.6 ng/mL, and 5.2 pg/mL, respectively. These results demonstrate that the development of reproducible SERS substrates is a critical technology for high-throughput multiplex analysis of various biomolecules [38].

Das et al. developed a SERS-based immunosensor platform for multiplex detection by fabricating microarray chips with gold nanopopcorn nanostructures as substrates, enabling the simultaneous diagnosis of the acute febrile diseases scrub typhus and murine typhus (Figure 4b). The platform targeted IgG and IgM antibodies specific to the pathogens causing each disease, using these antibodies as biomarkers. SERS signals were generated upon the antibodies binding to their respective target antigens by the formation of complexes with gold nanoparticles labeled with malachite green isothiocyanate (MGITC), designed to enhance the Raman signal. To detect the antibodies, target antibody titers in serum were serially diluted to determine the LODs, and thus the concentrations are expressed in terms of dilution factors. The LODs for *Orientia tsutsugamushi* IgG/IgM were 1:20.4 and 1:7.03, respectively, while for *Rickettsia typhi* IgG/IgM, the LODs were 1:16.8 and 1:12.5, respectively [221].

Cao et al. developed a uniform Au nanocrown array (AuNCA), which provides enhanced SERS signals for the simultaneous detection of CRC biomarkers (Figure 4c). AuNCA was functionalized by hybridizing the aptamer specific to the target protein and ssDNA with the Raman reporter molecules, Cy5 and 5-FAM. In the presence of target proteins in the sample, the aptamers selectively bound to the targets, leading to the release of the ssDNA from the surface. This triggered a decrease in the SERS signal, enabling quantitative detection via a signal-off, one-step recognition–release mechanism. Using this platform, two CRC biomarkers, hnRNP A1 and S100P, were sensitively and specifically detected within 15 min, with LODs of 0.031 pg/mL and 0.057 pg/mL, respectively. In addition, clinical analyses were performed using 30 serum samples from healthy individuals and 30 serum samples from CRC patients. Compared with gold-standard ELISA methods, correlation coefficients of 0.982 and 0.987 were obtained [8]. These studies show that multiple disease biomarkers can be detected precisely and reliably while ensuring reproducibility of SERS substrates, greatly expanding the possibility of early diagnosis and customized treatment.

### 4.2. SERS Nanotag-Based Multiplex Biosensor

**Figure 5:**
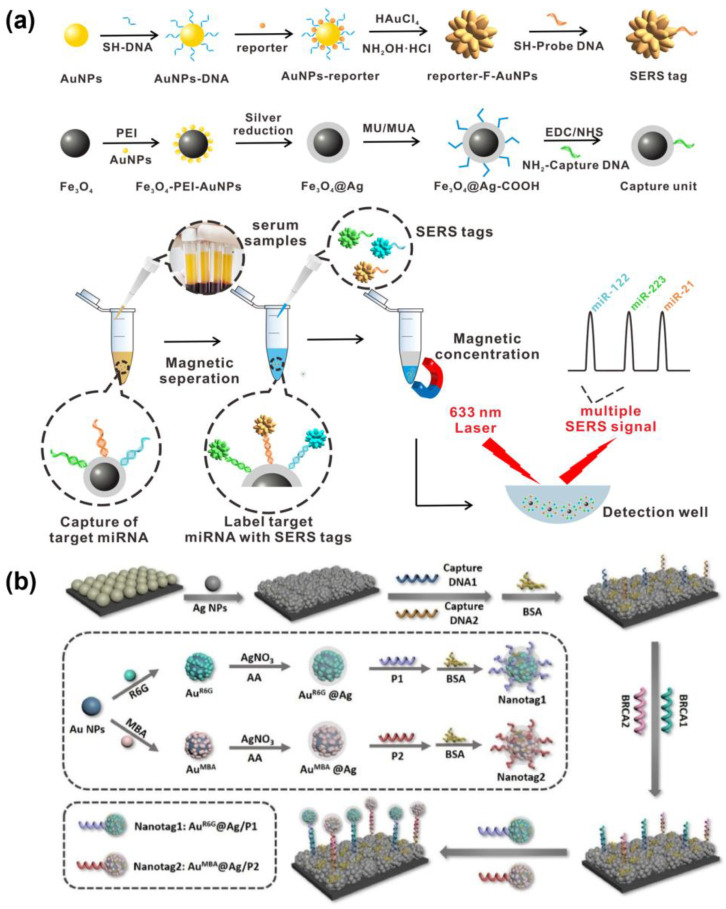
SERS nanotag based multiplex detection platforms. (**a**) Schematic illustration of an ultrasensitive and multiplex SERS biosensor for simultaneous detection of multiple microRNAs (miRNAs) using fractal gold nanotags for early diagnosis and prognosis of HCC. DNA-engineered SERS tags were prepared by modifying three probe DNAs (complementary to miR-122, miR-223, and miR-21) with distinct Raman reporters (R6G, CV, and 4-ATP). These tags hybridized with captured targets on AgMNPs to form sandwich structures, whose SERS signals were measured after magnetic separation. Reproduced with permission from [222]. Copyright 2021, American Chemical Society. (**b**) Schematic representation of dual-biomarker detection using SERS nanotags for ultrasensitive breast cancer diagnosis. Capture DNAs immobilized on the PS@Ag SERS substrate hybridize with target DNAs, which in turn bridge to probe DNAs on SERS nanotags (Au@Ag-R6G/P1 and Au@Ag-MBA/P2). This sandwich assembly facilitates highly sensitive SERS signal detection. Reproduced with permission from [224]. Copyright 2025, Elsevier.

Wu et al. proposed a magnetically assisted sandwich-type SERS-based biosensor for the ultrasensitive and multiplex detection of miRNA biomarkers associated with HCC (Figure 5a). The system employed three uniform, star-shaped fractal AuNPs (F-AuNPs) with core–shell structures encoded with DNA-engineered Raman dyes, each producing distinct Raman signals. These SERS tags were functionalized with probe DNAs specific to miRNA-122, miRNA-223, and miRNA-21, which were labeled with rhodamine 6G (R6G), crystal violet (CV), and 4-aminothiophenol (4-ATP), respectively, to form sandwich complexes. In this system, Ag-coated Fe_3_O_4_ magnetic nanoparticles (AgMNPs) functionalized with streptavidin were conjugated to the biotinylated capture probe within the sandwich structure, enabling magnetic enrichment and stable coupling of the capture substrate/miRNA/SERS tag complex. This conjugation facilitated simultaneous detection of miRNA-21, miRNA-122, and miRNA-223 through their unique Raman signatures. The recorded LODs were as low as 311 aM, 349 aM, and 374 aM, respectively, demonstrating excellent selectivity, high sensitivity, and accuracy. This platform successfully detected all three miRNAs in actual clinical serum samples, highlighting the strong potential of this platform for early cancer diagnosis and disease monitoring [222].

Shim et al. published another study focusing on high-sensitivity, multiplex SERS detection. This research proposed a multiplex digital sensing platform based on bumpy core–shell (BCS) SERS nanoprobes. These unique BCS SERS nanoprobes were synthesized by first forming a self-assembled monolayer of the respective Raman reporter on a core Au nanoparticle. Next, a bumpy shell was grown, and the Raman reporters were re-attached to the outer surface of the BCS nanoprobe for multiplex detection of Aβ42 and Aβ40, which are diagnostic biomarkers for Alzheimer’s disease (AD). Since the characteristic peaks of the 2,3,5,6-tetrafluoro-4-mercaptobenzoic acid (TFMBA) BCS and 4-mercaptobenzoic acid (4-MBA) BCS nanoprobes were distinguishable, the SERS signals for both Aβ42 and Aβ40 were selectively detected without interference. Specifically, an anti-Aβ16 antibody enabling capture of both targets was immobilized on the substrates, and only presence of the specific antigen allowed for BCS binding. The LODs showed excellent performance at 87 ag/mL and 1.0 fg/mL, respectively [223].

Ge et al. developed a SERS-based lab-on-a-chip (LoC) system incorporating a multi-signal amplification strategy for the early diagnosis of Parkinson’s disease (PD). Gold nanobipyramids (GNBPs) were functionalized with Raman reporter molecules and hairpin-structured DNA1 (hDNA1) to serve as SERS nanotags. When the target is present, the sequence-specific strand displacement amplification (SDA) reaction is initiated, releasing the trigger DNA. This trigger then induces a CHA between the SERS nanotags and magnetic beads, leading to the aggregation of GNBPs on the surface of the magnetic beads. This generated hotspot allows for sensitive detection of miR-214 and miR-221 and enables highly selective detection based on sequence hybridization. The respective LODs showed excellent sensitivity with 5.14 aM and 5.92 aM, and such high-sensitivity detection has great potential as an early diagnosis platform. Additionally, the sensing performance of the chip was first validated using a PD mouse model, and subsequent verification was performed with actual human samples. Using blood samples from 30 PD patients and 30 healthy individuals, a high agreement with the gold-standard qRT-PCR method was observed, with a relative error of less than 6.85% [219].

Core–shell nanoparticles provide advantages such as the chemical stability of the core, plasmonic enhancement of the shell, and efficient surface functionalization. Mousavi et al. leveraged these properties to develop a paper-based SERS platform for on-site multiplex detection of cancer biomarkers, employing Au–Fe_3_O_4_ core–shell nanoparticles conjugated with target-specific antibodies and distinct Raman reporters. The system enabled interference-free simultaneous detection of CEA and neuron-specific enolase (NSE), achieving low detection limits of 0.9 pg/mL and 1.6 pg/mL with high recovery rates in serum samples [218]. Moreover, Hou et al. utilized Au@Ag core–shell nanoparticles in a SERS nanotag platform for ultrasensitive detection of breast cancer gene 1 (BRCA1) and BRCA2 (Figure 5b). Target-specific oligonucleotide probes and unique Raman reporters induced nanotag aggregation upon hybridization, producing intense SERS signals with detection limits of 0.61 pM for BRCA1 and 0.78 pM for BRCA2, demonstrating strong potential for clinical diagnostics [224].

Despite extensive efforts to improve reproducibility, the inherent heterogeneity of nanoscale architectures and the unpredictable interactions within complex samples continue to hinder the quantitative reliability and commercial viability of SERS-based techniques [225,226]. Achieving high-resolution analysis of SERS signals requires the use of precise light sources and advanced spectroscopic systems; however, this requirement may impose limitations relating to instrumentation complexity and initial cost, ultimately restricting practical applications [227]. A detailed list of representative SERS-based multiplex biomarker detection platforms is presented (Table 2).

**Table 2:** Applications of SERS-based Multiplex Biomarker Detection.

Target Biomarkers	Nanoparticles	Method Performance	Sample	Ref.
CK-MB, Myoglobin, and cTnI	Gold–silica multilayered pyramidal plasmonic metasurface substrate	LOD: 0.04 ng/mL, 3.6 ng/mL, and 5.2 pg/mL Linear range: 0.1–300 ng/mL, 6–4000 ng/mL, and 8–567 pg/mL	Mixtures of serum	[38]
*O*. *tsutsugamushi* IgG and IgM, *R*. *typhi* IgG and IgM	Microarray chips with gold nanopopcorn nanostructures	LOD: 1:20.4 and 1:7.03, 1:16.8 and 1:12.5 RSD: 5.46%, 4.41%, 4.87%, and 10.15% (Antibody titers)	Human serum (Healthy for negative; Diluted patient sample for positive)	[221]
hnRNP A1, S100P	Uniform Au nanocrown array (AuNCA)	LOD: 0.031 pg/mL and 0.057 pg/mL Linear range: 1 pg/mL–1 µg/mL Correlation coefficients: 0.982 and 0.987	Real clinical serums from healthy and patients	[8]
miRNA-21, miRNA-122, miRNA-223	Star-shaped fractal AuNPs (F-AuNPs) and silver magnetic nanoparticles (AgMNPs)	LOD: 311 aM, 349 aM, and 374 aM Linear range: 1 fM–10 nM	Actual clinical serum samples	[222]
Aβ42, Aβ40	Bumpy core–shell (BCS) SERS nanoprobes	LOD: 87 ag/mL and 1.0 fg/mL RSD: <3%	Human blood plasma and aCSF	[223]
miR-214, miR-221	Gold nanobipyramids (GNBPs)	LOD: 5.14 aM and 5.92 aM Relative error compared with ELISA: <6.85%	Human blood samples from healthy and patients	[219]
CEA, NSE	Au–Fe_3_O_4_ core–shell nanoparticles	LOD: 0.9 pg/mL and 1.6 pg/mL	-	[218]
BRCA1, BRCA2	Au@Ag core–shell nanoparticles	LOD: 0.61 pM and 0.78 pM Linear range: 1 pM–1 µM RSD: 4.75%	-	[224]

## 5. Colorimetric Multiplex Biomarker Detection

Colorimetric assays have been widely employed for biomarker detection in disease diagnostics due to their simplicity and intuitive visual readout [228,229]. Colorimetric methods are highly suitable for POC testing, owing to advantages such as low cost and minimal reliance on instrumentation [6,230]. Moreover, certain approaches can achieve distinct colorimetric responses solely through nanoparticles, bypassing the requirement for enzymes or chromogenic substrates, thereby providing potential advantages in terms of cost effectiveness and storage stability [231]. For multiplex detection of targets using colorimetric methods, it is generally necessary to employ substances with distinct colors or to apply spatial coding strategies that generate target-specific signals [232]. The rapid and straightforward nature of colorimetric detection not only enables such multiplexing but also enhances diagnostic efficiency and accessibility [233,234]. Common strategies for inducing color changes in such systems include aggregation of AuNPs [12,235], the use of core–shell nanoparticles or metal NP-assembled silica nanoparticles [236], and measurement of the colorimetric reaction of 3,3′,5,5′-tetramethylbenzidine (TMB) catalyzed by nanozymes [237]. Since the naked eye can discern signal readouts, colorimetric sensing is frequently integrated with paper-based platforms or microfluidic chips for on-site diagnostics [238,239].

These drawbacks make performing colorimetric multiplex detection in colloidal systems quite challenging. Nevertheless, several studies have achieved multiplex detection by employing two particles with distinct absorption peaks. Indeed, Su et al. developed a dual colorimetric amplification biosensor for the multiplexed detection of miRNAs using magnetic beads (MBs), AuNPs, and G-quadruplex/hemin DNAzymes. In this system, miR-10b initiates a CHA between AuNPs and MBs, promoting their aggregation and enabling facile magnetic separation. Ultimately, the fading of the red color of AuNPs indicates the presence of the target. Meanwhile, miR-21 induces a hybridization chain reaction (HCR) on the MB surface, which, when K^+^/hemin is added, forms a G-quadruplex/hemin DNAzyme that catalyzes the ABTS/H_2_O_2_ reaction, resulting in a green color change. The system demonstrated LODs of 1.5 nM for miR-10b and 2.2 nM for miR-21 [240]. However, verification of the two colors required magnetic separation as an additional procedure. Zhang et al. reported a virus-target multiplex detection system that simultaneously employed two types of AuNPs, gold nanospheres (GNSp) and gold nanoshells (GNSh), which possess distinct absorption peaks and appear red and blue, respectively. Nucleotides specific to the *M* gene in the IAV and the *E* gene in SARS-CoV-2 were immobilized on the surfaces of GNSp and GNSh, enabling particular binding to their respective targets [236]. Under salt-induced conditions, gold nanoparticles easily aggregate due to weak electrical repulsion between particles. However, when oligonucleotides on the surface of functionalized gold nanoparticles combine with the target sequence, the particles stabilize and nanoparticle aggregation is suppressed. Therefore, nanoparticle aggregation was prevented in the presence of the target, thus maintaining the characteristic color of each particle type. Using the change in plasmon resonance of mixed nanoparticles, we achieved a LOD of 33 nM for GNSp and 10 nM for GNSh. Such colloidal colorimetric multiplexing with nanoparticles is limited by the reliance of the platform on UV–Vis spectrophotometer readouts for accurate color discrimination [241].

Colorimetric detection is well suited for integration with paper-based sensing systems, such as LFAs, which, due to the advantage of enabling visual result interpretation, represent promising POC tools. Several studies have reported detection strategies that induce target-specific reactions directly on LFA strips, while multiplex detection using such platforms has also been investigated [239]. AuNPs are widely employed to generate color signals on the lines of LFA strips; however, multiplexing is limited when using only a single type of metallic nanoparticle. Hence, strategies have been developed to functionalize nanoparticles with different probes and guide these nanoparticles to bind at distinct positions on the strip [16], or to design probes with multiple colors and relatively uniform size or morphology so that different colors can be displayed at the same position [17].

### 5.1. Paper-Based Multiplex Colorimetric Biosensor

**Figure 6:**
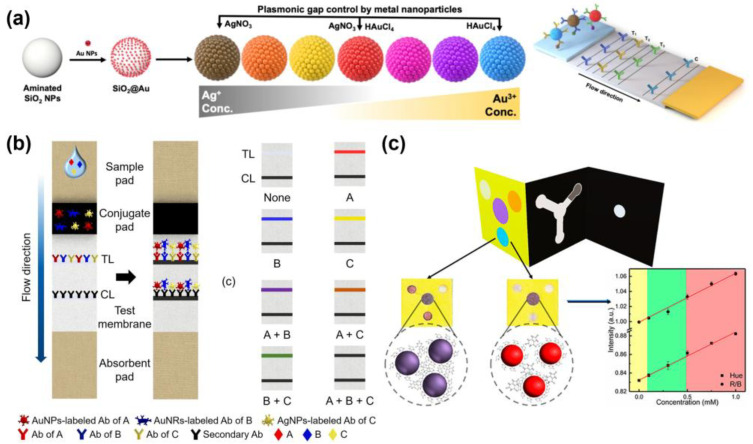
Multiplex biomarker detection using paper-based colorimetric methods. (**a**) Schematic illustration of the fabrication of seven multicolored PINs, with tunable nanogaps and distinct optical colors, and the LFA system using three PINs. Different probe colors correspond to distinct target biomarkers and enable visual discrimination in the test zone. Reproduced with permission from [17]. Copyright 2024, Springer Nature. (**b**) Schematic diagram of a multiplex LFIA system using gold nanostructures. Detection of the presence of three distinct targets through color differentiation on a single test line. Reproduced with permission from [238]. Copyright 2024, Springer Nature. (**c**) Schematic diagram of microfluidic paper-based analytical devices (μPADs) using AuNPs for multiplex detection. Distinct colors in each detection zone indicate individual analyte recognition events. Reproduced with permission from [231]. Copyright 2021, American Chemical Society.

Shin et al. developed a highly sensitive multiplexed colorimetric lateral-flow immunoassay based on plasmon-controlled metal–silica isoform nanocomposites (PINs) with tunable multicolor plasmonic properties (Figure 6a). Au nanoseeds were immobilized to SiO_2_ nanoparticles, and Ag, Au, or both metals were grown via seed-mediated growth methods to produce PINs exhibiting seven distinctly distinguishable colors. After synthesizing the nanoparticles, the extinction spectra of the subsequent nanoparticles were analyzed to confirm the growth-dependent optical characteristics. Furthermore, the fabricated brown, navy, and red PINs were employed to simultaneously detect key biomarkers of gastric, pancreatic, and prostate cancers, specifically, intercellular adhesion molecule 1 (ICAM1), carbohydrate antigen 19-9 (CA19-9), and prostate-specific antigen (PSA), respectively. The LODs were determined as 6.65 ng/mL, 0.04 U/mL, and 0.12 ng/mL, demonstrating the capability of the assay to sensitively detect biomarker concentrations well below the pathological ranges for each disease [17].

Moon et al. developed a lateral flow immunoassay (LFIA) system in which red AuNPs, yellow AgNPs, and cyan AuNRs were synthesized and engineered to flow simultaneously within a single LFIA device, enabling multiplexed detection through the analysis of a single test line color (Figure 6b). Different nanoparticle types were used to calculate the respective average friction coefficients, and the physical characteristics of the LFA strip, including porosity and penetration volume, were optimized. The system allowed for the simultaneous detection of key biomarkers, AFP for liver cancer, NSE for lung cancer, and CEA for various cancers, producing eight distinct test line colors corresponding to the presence or absence of each target. This approach demonstrated effective combinatorial color responses and achieved sensitive detection of single and multiple biomarkers with a LOD of 50 ng/mL [238].

Pinheiro et al. developed microfluidic paper-based analytical devices (μPADs) incorporating AuNPs for the multiplex detection of glucose, uric acid, and cholesterol, which are key biomarkers for monitoring diabetes and the respective complications (Figure 6c). The device was assembled by folding three paper sheets, enabling the combination of horizontal and vertical fluid flows to generate uniform colorimetric signals. For glucose detection, AuNPs were synthesized in situ on paper substrates, whereas the loads of glucose, uric acid, and cholesterol were 1.25 mM, 71 μM, and 81 μM, respectively [231].

The colorimetric detection method has limitations in terms of sensitivity and precision, and various studies are being conducted to detect low-concentration analytes [242,243,244]. The next-generation technology that enables the quantification and multi-detection of colorimetric signals using high-resolution cameras and artificial intelligence (AI)-based image analysis of smartphones has recently been actively studied [245,246,247,248,249].

Wang et al. developed a paper-based colloidal gold multi-vertical flow assay to rapidly detect three acute kidney injury (AKI) biomarkers: Neutrophil Gelatinase Associated Lipocalin (NGAL), Cystatin C (CysC), and Retinol-Binding Protein 4 (RBP4). In this system, specific capture antibodies were preimmobilized at four spatially separated immune response sites on the sensing membrane. AuNPs were bound to detect antibodies by colorimetric markers. The resulting images were able to be read qualitatively to the naked eye or quantitatively using a smartphone-based reader. The three AKI biomarkers could be detected simultaneously within 10 to 15 min. The LODs of NGAL, CysC, and RBP4 were 1.04 ng/mL, 0.96 ng/mL, and 1.17 ng/mL, respectively. Specifically, the detection sensitivity and specificity of clinical serum specimens taken from 25 AKI patients and 25 healthy individuals were measured as 92% and 88%, respectively [245].

In the case of the study by Wang et al., a portable multiplexed 3D-fold pad was fabricated to achieve visualized, accurate, and simultaneous detection of adrenaline and glucose. Multiple colorimetric analyses of adrenaline and glucose were performed using Ag-doped copper sulfide nanoparticles (Cu_1-X_Ag_X_SNP) as a multifunctional sensing probe. Adjustable lacase-like and peroxidase-like activities of Cu_1−X_Ag_X_SNP produced orange-red and blue, respectively, in the adrenaline and glucose detection regions of μPAD. A 3D folded μPAD can improve color uniformity by controlling the vertical supply of analytical solution with the sensing region. Color signals were converted to RGB values through a smartphone application, and fast and accurate result analysis was performed. The LODs of adrenaline and glucose were 10.2 nM and 11.5 μM, respectively [246]. Smartphone-based colorimetric multi-detection technology has the potential to evolve into a powerful platform that can quickly and accurately identify multiple pathogens simultaneously in the field without the need for separate expensive equipment.

### 5.2. Dual-Mode, Including Colorimetric Methods for Multiplex Detection

Limitations remain in colorimetric-based multiplex detection compared to other detection techniques in terms of sensitivity and an inability to perform quantitative analysis [248]. Therefore, dual-mode systems have been reported in which qualitative analysis is first conducted visually using colorimetry, followed by sensitive quantitative analysis using auxiliary readout methods such as fluorescence or Raman spectroscopy [236,237].

**Figure 7:**
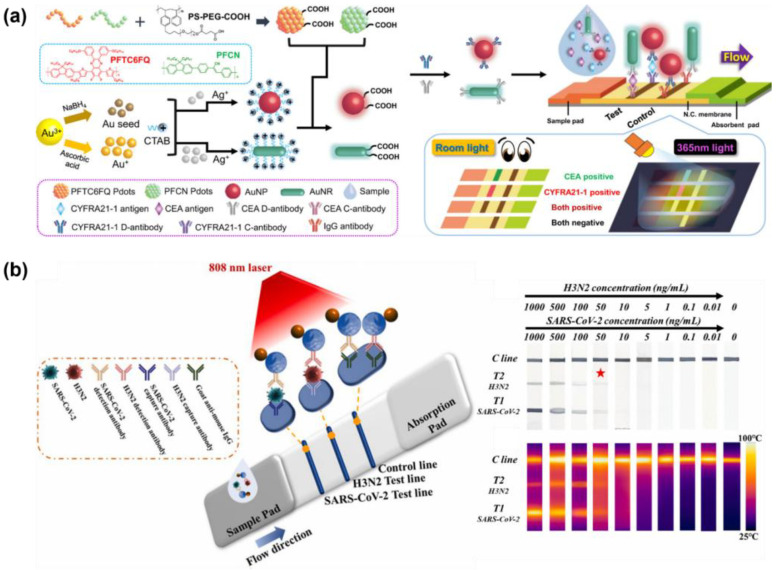
Multiplex biomarker detection using dual-mode, including colorimetric methods. (**a**) A schematic diagram of applications for multiple detection of CEA and CYFRA21-1 in fluorescent and colorimetric dual-mode LFA strips. AuNPs and AuNRs capped with PFTC6FQ and PFCN Pdots were conjugated with antibodies against CEA and CYFRA21-1 for antigen detection. The results were visualized either by color change on the test line or by fluorescence under UV illumination. Reproduced with permission from [249]. Copyright 2021, American Chemical Society. (**b**) Schematic illustration of dumbbell-structured Au_shell_–Fe_3_O_4_ nanoparticles modified with target-specific antibodies for multiplex viral antigen detection on a LFIA strip (Left). Dual-mode strip images for quantification of both targets, the upper image shows results obtained by visual inspection, while the lower image shows measured temperature changes (Right). Reproduced with permission from [250]. Copyright 2025, Elsevier.

Yang et al. developed Au@Pdot nanoparticles. In prior work by Yang et al., the authors demonstrated that fluorescent semiconducting polymer nanoparticles (Pdots) exhibit high fluorescence intensity, excellent colloidal stability, and facile surface engineering for bioconjugation (Figure 7a). Building on this, Yang and co-authors integrated Pdots into an immunochromatography test strip (ICTS) platform to construct a POC system capable of multiplexed antigen detection. The system employed a dual-mode approach, allowing for qualitative visual determination of antigen presence while simultaneously enabling quantitative measurement of target concentrations via fluorescence. Two types of Pdots, poly(2,7-(9,9-dioctylfluorene)-alt-2,3-diphenylacrylonitrile) (PFCN) and poly(9,9-dioctylfluorene-co-2,3-bis(3-(hexyloxy)phenyl)-5,8-di(thiophen-2-yl)quinoxaline) (PFTC6FQ), were selectively coated onto the surfaces of AuNRs and AuNPs, respectively. Using this system, the LODs for the cytokeratin 19 fragment (CYFRA21-1) and CEA lung cancer biomarkers were determined to be 0.07 ng/mL and 0.12 ng/mL, respectively. This allows for both qualitative and quantitative detection of non-small-cell lung cancer (NSCLC) using only a single drop of whole blood within 15 min [249].

Wen et al. developed a LFIA system for the colorimetric and photothermal detection of respiratory viruses requiring early diagnosis due to similar clinical symptoms, specifically H3N2 influenza and SARS-CoV-2 (Figure 7b). To exploit AuNPs for both colorimetric and photothermal effects, the LSPR peak of the nanoparticles should be carefully tuned to align with the NIR irradiation light. Therefore, the authors developed dumbbell-shaped nanoparticles combining AuNPs and Fe_3_O_4_, which exhibited a high photothermal conversion efficiency due to the synergistic interaction between the two materials. As the thickness of the Au shell decreased, the LSPR absorption peak shifted to 805 nm. The system visually detected both H3N2 and SARS-CoV-2 antigens with an LOD of 50 ng/mL. Moreover, the LODs photothermal detection on the same strip were calculated as 2 pg/mL and 7 pg/mL, respectively, and the sensitivity was approximately10,000 times better than the colorimetric results. The results confirm that the dual-mode system effectively overcame the sensitivity limit of conventional colorimetric detection [250].

Colorimetric-based multiplex biomarker detection has been actively studied due to its recognized potential in POCT; the requirement for rapidity and cost effectiveness is critical [229,251]. Nevertheless, several challenges, such as low sensitivity and quantitative accuracy for detecting trace analytes, remain at early disease stages or in applications demanding high analytical precision. Despite these limitations, the inherent simplicity and accessibility of colorimetric detection highlight its promise, warranting further advanced investigations aimed at enhancing its application for POCT. A detailed list of representative colorimetric-based multiplex biomarker detection platforms is presented (Table 3).

## 6. Conclusions and Future Perspectives

Optical-based multiplex detection systems have showed remarkable performance by leveraging various nanomaterials for sensitive and simultaneous biomarker detection. AuNPs and AgNPs provide plasmonic signal enhancement and efficient surface functionalization, forming highly reproducible “hotspots” for SERS and fluorescence-based detection of proteins and nucleic acids. Two-dimensional nanomaterials such as GO efficiently quench fluorescence, with fluorescence restored upon target binding, enabling interference-free multiplex detection. These platforms allow for the multiplex detection of protein and nucleic acid biomarkers with high sensitivity and selectivity. Colorimetric systems, while more limited by the need for distinct absorption spectra or additional separation steps, provide rapid, visually interpretable signals and can be quantified spectrophotometrically, demonstrating potential for POC applications.

Despite these advances, several challenges remain. Although many studies have evaluated their systems using clinical serum samples, most were performed under controlled laboratory conditions, and robust performance in complex biological matrices remains challenging. Translating these technologies into clinical practice involves multiple steps, including preclinical studies, clinical trials, and regulatory approval, beyond simply testing with clinical samples. Addressing nonspecific adsorption, background interference, and reproducibility issues is essential, and the number of simultaneously detectable targets is often constrained by spectral overlap and excitation requirements.

Future directions include expanding multiplexing capacity through the combinatorial use of distinct optical reporters, tunable nanomaterials, and innovative encoding strategies. Additional integration with microfluidic platforms, automated sample handling, and portable detection devices is critical for realizing POC and real-time applications. Continued optimization of optical-based multiplex detection systems promises to accelerate biomarker discovery, enable precise disease diagnosis, and support personalized medicine by providing rapid, sensitive, and multiplexed analytical capabilities. Addressing these challenges requires the establishment of standardized protocols to ensure reproducibility and reliability, as well as interdisciplinary cooperation between materials science, biotechnology, and clinical medicine. In addition, advances in signal analysis and data integration using artificial intelligence are expected to spur the transition of SERS and fluorescent/color biosensors into practical diagnostic solutions.

In particular, AI- and smartphone-based signal processing and data fusion technologies are key tools for the quantitative and reproducible interpretation of complex multi-marker patterns and are expected to play an important role in achieving both diagnostic accuracy and standardization in the future. Furthermore, advances in these technologies, when combined with portable devices and user-friendly interfaces, are expected to significantly increase usability in field diagnostics and telemedicine environments.

## Figures and Tables

**Table 1:** Applications of Fluorescence-based Multiplex Biomarker Detection.

Target Biomarkers	Nanoparticles	Method Performance	Sample	Ref.
miR-21, miR-141	pAuNRs	LOD: 0.1 pM	Human sample (5%)	[37]
SARS-CoV-2 spike protein, Influenza A (H1N1) hemagglutinin, RSV fusion protein, Adenovirus hexon protein	PDDA-conjugated polystyrene NPs on Ag island substrate (pSilverF)	LOD: 0.168 NCU/mL, 0.023 NCU/mL and 0.168 NCU/mL	Human serum (42 patient samples, 26 healthy controls)	[191]
miR-21, miR-122	AuNPs with hairpin DNA probes + EDC DNA logic circuit	LOD: 2.4 × 10^5^ to 6.8 × 10^6^ particles/μL Sensitivity: 93.3%, Accuracy: 93.3%, AUC: 0.92	Exosomes derived from HCC patient serum	[192]
MCP-1, IL-6, IL-10, IL-3, IL-1β, TNF-α	Au nanodimple substrate decorated with AuNPs and TSA amplification	LOD: <1 pg/mL 100% accuracy	Clinical plasma samples 100% accuracy	[193]
miR-21, miR-141	CDs or QDs (fluorescent reporters) and GO quencher	LOD: 60 pM, 50 pM	Human serum	[202]
miR-21, miR-141	CQD-labeled probes and GO quencher	LOD: 4.7 fM Recoveries: 93.3–109.2%	Human serum samples	[203]
miR-21, miR-10b	Au nanowire/GO nanomotors with multicolor QD-labeled ssDNA probes	LOD: 0.5–10 pM OFF–ON switching within 15 min	Intracellular detection in living cells	[204]
AFP, DKK1, GPC3	CdSe/ZnS core–shell QDs (525, 585, 625 nm) conjugated with specific antibodies	LOD: 0.625 ng/mL, 1.25 ng/mL and 2.5 ng/mL	Mixed reference antigen sample	[207]
miR-135b, miR-21, miR-96	WSNs with QD–DNA	LOD: 5 fM, 19 fM and 8 fM Linear range: 10 fM–100 nM RSD: <5%	Clinical serum samples	[205]
miR-33, miR-125b	Dual QDs (donors) conjugated with ssDNA and BHQ-functionalized multifunctional nanomaterials (quencher)	LOD: 0.06 nM, 0.02 nM RSD: <2.1%	Human serum samples	[206]
CA125, CEA, AFP	Porous silica-based PhC beads (dSiO_2_) functionalized with antibodies and CdTe QDs	LOD: 0.52 ng/mL, 0.64 ng/mL and 0.79 U/mL RSD: <5.5%	Human serum samples	[208]
HPV16, HPV18, HPV33	Bioinspired PhC barcodes: Structural colors at 418 nm, 520 nm, 652 nm	LOD: 0.025 pM	Synthetic nucleic acids	[211]
SARS-CoV-2 N gene, IAV, IBV	PhC barcodes encapsulated in porous hydrogel	LOD: 200 aM Recovery rates range: 88.9–112.6%	Clinical RNA samples	[210]
miR-155, miR-let-7a	Luminescence-quenched UCNP–microbead assemblies (green 541 nm, blue 475 nm) with quencher-labeled reporters	LOD: 17 aM, 22 aM	Single-cell analysis	[211]
SARS-CoV-2 ORF1ab gene, N gene	UCNPs and Ti_3_C_2_T_x_ MXene quencher	LOD: 15 fM, 194 fM	Spiked samples	[212]

**Table 3:** Applications of Colorimetric-based Multiplex Biomarker Detection.

Principle	Target Biomarkers	Nanoparticles	Method Performance	Sample	Ref.
Colorimetric	miR-10b, miR-21	MBs, AuNPs, G-quadruplex/hemin DNAzyme	LOD: 1.5 nM, 2.2 nM Spike recoveries: 93.3% to 109.2% RSDs: 2.1% and 7.2%	Spiked into the diluted healthy human serum sample	[240]
	*M* gene in the influenza A virus, *E* gene in SARS-CoV-2	GNSp, GNSh	LOD: 33 nM, 10 nM	-	[241]
	ICAM1, CA19-9, PSA	Plasmon-controlled metal–silica isoform nanocomposites (PINs)	LOD: 6.65 ng/mL, 0.04 U/mL, 0.12 ng/mL	-	[17]
	AFP, NSE, CEA	AuNPs, AgNPs, AuNRs	LOD: 50 ng/mL (each)	-	[238]
	Glucose, uric acid, cholesterol	AuNPs	LOD: 1.25 mM, 71μM, 81μM	-	[231]
	NGAL, CysC, RBP4	AuNPs	LOD: 1.04 ng/mL, 0.96 ng/mL, 1.17 ng/mL Sensitivity: 92% Specificity: 88%	Clinical serum samples	[245]
	Adrenaline, Glucose	Cu_1−x_Ag_x_ S NP	LOD: 10.2 nM, 11.5 μM Linear range: 0.05–30 μM, 0.02–12 mM	Real sample	[246]
Dual (Colorimetric and fluorescence)	CYFRA21-1, CEA	Au@Pdot, AuNRs, AuNPs	Cutoff value: 3.3 ng/mL, 5 ng/mL (Colorimetric) 0.07 ng/mL, 0.12 ng/mL (Fluorescence) Specificity: 95%	Clinical serum sample	[249]
Dual (Colorimetric and photothermal effects)	H3N2 influenza, SARS-CoV-2	Janus Au_shell_–Fe_3_O_4_	LOD: 50 ng/mL (Each) (Naked eye), 2 pg/mL, 7 pg/mL (Photothermal effects)	-	[250]
